# Preliminary Research on Dietary Supplementation of Potassium Magnesium Sulphate on Transport Stress in Finishing Pigs Prior to Slaughter

**DOI:** 10.3390/ani15030362

**Published:** 2025-01-27

**Authors:** Bailei Cui, Yunxia Xiong, Xiaolu Wen, Shengnan Wu, Yi Huang, Hao Xiao, Shuting Cao, Zongyong Jiang, Li Wang, Shenglan Hu

**Affiliations:** 1 State Key Laboratory of Swine and Poultry Breeding Industry, Key Laboratory of Animal Nutrition and Feed Science in South China, Ministry of Agriculture and Rural Affairs, Guangdong Key Laboratory of Animal Breeding and Nutrition, Lingnan Modern Agricultural Science and Technology Guangdong Provincial Laboratory Maoming Branch, Institute of Animal Science, Guangdong Academy of Agricultural Sciences, Guangzhou 510640, China; 18776703467@163.com (B.C.); xiongyunxia@gdaas.cn (Y.X.); wenxiaolu@gdaas.cn (X.W.); caoshuting@gdaas.cn (S.C.);; 2 College of Animal Science and Technology, Guangxi University, Nanning 530004, China

**Keywords:** potassium magnesium sulphate, transport stress, finishing pigs, meat quality, stress response

## Abstract

Transport stress prior to slaughter frequently induces a stress response, negatively affecting meat quality. This study investigated the impact of dietary potassium magnesium sulphate (PMS) supplementation during the fattening stage on the stress response and meat quality in finishing pigs subjected to transport stress. The experiment involved two phases. Initially, 48 finishing pigs (68.00 ± 0.40 kg) were randomly allocated into two groups: a control group receiving a basal diet (CON) and a PMS-supplemented group receiving the basal diet with 0.50% PMS. Each group was housed in six pens, with four pigs per pen. After 60 days of feeding, in the second phase, two pigs from each pen were randomly selected for slaughter, with one pig subjected to a 4 h transportation stress prior to slaughter. Pigs were categorized into four treatment groups based on diet and stress: (1) control without transport stress, (2) control with transport stress, (3) PMS-supplemented without transport stress, and (4) PMS-supplemented with transport stress. Serum, jejunum, and *longissimus thoracis* muscle (LM) samples were collected. The results indicated that dietary PMS supplementation did not significantly affect growth performance during the fattening stage (*p* > 0.05). However, following transport, the PMS pigs showed a reduction in norepinephrine and cortisol concentrations (*p* = 0.09, *p* < 0.05) and a significant increase in serum glutathione peroxidase (GSH-Px) activity (*p* < 0.05). Furthermore, PMS supplementation significantly increased serum catalase (CAT), total antioxidant capacity (T-AOC), alkaline phosphatase (AKP) activity, and high-density lipoprotein cholesterol (HDL-C) levels (*p* < 0.05), while significantly reducing cholesterol (CHO) levels (*p* < 0.05). Transport stress adversely affected the intestinal health of finishing pigs, as evidenced by a decrease in intestinal villus height (0.05 < *p* < 0.1), a condition ameliorated by PMS supplementation. Additionally, transported pigs exhibited a higher drip loss_24h_ in LM (*p* < 0.05), which was also alleviated through PMS supplementation. In conclusion, PMS supplementation mitigates transport stress and improves meat quality in finishing pigs.

## Simple Summary

Transport stress prior to slaughter frequently induces a stress response, compromising meat quality. The aim of this study was to investigate the effect of dietary potassium magnesium sulphate (PMS) supplementation during the fattening stage on the stress response and meat quality in finishing pigs subjected to a transport challenge prior to slaughter. Our findings indicate that while dietary PMS supplementation did not affect growth performance during the fattening period, it significantly enhanced antioxidant capacity and meat quality. In the transportation model, PMS supplementation significantly improved the antioxidant capacity, stress response and meat quality of finishing pigs. These results suggest potential applications of PMS in the livestock industry to enhance animal welfare and meat quality.

## 1. Introduction

In the modern breeding industry, it is common for raising facilities and slaughterhouses to be geographically separated, often by a considerable distance. The transportation of animals from farms to slaughterhouses is predominantly conducted using vehicles. However, this adversely affects the welfare, physiology, and carcass quality of slaughtered pigs [1]. Many countries and regions have specific laws and regulations to protect animal welfare during transportation, e.g., laws in the European Union include requirements for transportation conditions, such as temperature control, ventilation and feed, in order to ensure animal welfare and avoid unnecessary suffering during transportation. However, during transportation, factors such as noise, dehydration, overcrowding, stampedes, and temperature fluctuations may still induce various physiological and psychological injuries in pigs [2,3], resulting in compromised meat quality and intestinal damage [4]. It has been reported that transportation leads to increased drip loss, decreased muscle pH value at 45 min post-mortem, and reduced antioxidant capacity, all of which negatively affect meat quality [5,6]. During transportation, the stress response significantly elevates reactive oxygen species (ROS) levels in pigs, directly contributing to oxidative stress [7]. This oxidative stress can disrupt normal physiological functions, leading to a decline in the quality of animal products [5,8]. Transport stress increases the likelihood of ectopic translocation of gut bacteria, thereby elevating morbidity and carcass damage [9,10].

To alleviate transportation-induced stress in pigs, dietary supplementation with vitamins, minerals, and amino acids has been demonstrated to reduce the adverse effects of the stress response [2,3,11]. Potassium, the third most abundant essential element after calcium and phosphorus, is vital for maintaining normal physiological functions [12]. It is primarily involved in the metabolism of carbohydrates, proteins, and energy, and is essential for maintaining osmotic pressure and acid–base balance in intracellular and extracellular fluids [13]. Magnesium, another crucial mineral for pigs, plays a significant role in various metabolic activities [14]. It acts as an enzyme activator, influences nerve impulse transmission and muscle tone maintenance, and exhibits antioxidant and stress resistance properties [15]. Additionally, dietary magnesium supplementation has been shown to enhance lifespan and antioxidant capacity in a mouse model of progeria [16].

Potassium magnesium sulphate (PMS) is a distinctive complex salt, naturally occurring from the combination of potassium sulphate and magnesium sulphate in the salt lake of Qinghai Province, China. Our previous study indicated that dietary supplementation with PMS enhanced the antioxidant capacity of weaned piglets [17]. However, the effects of PMS on finishing pigs, particularly regarding its potential to alleviate transportation-induced stress, have not been explored. Therefore, this study was carried out to investigate the effects of dietary supplementation with PMS on growth performance during the fattening period, as well as its effects on the stress response, antioxidant capacity, intestinal morphology and meat quality in finishing pigs subjected to transport stress. The findings are intended to provide insights for further application of PMS in the swine industry.

## 2. Materials and Methods

### 2.1. Animal Management and Experimental Design

The experiment was conducted in two phases. Initially, a total of 48 finishing pigs (Duroc × Yorkshire × Landrace) with an initial body weight (BW) of 68.00 ± 0.40 kg were selected and split into 2 groups: (1) a control group (CON) receiving a basal diet and (2) a PMS group (PMS) receiving the basal diet supplemented with 0.50% PMS. Each group was housed in six pens, with four pigs per pen (two barrows and two gilts). Pigs were fed twice daily at 8:00 and 15:00 and allowed access to feed and water ad libitum throughout the 60-day feeding trial. The BW of each pig was recorded at both the beginning and end of the experiment. Daily feed allocations and residuals in the feeders were also recorded. At the end of the experiment, average daily gain (ADG), average daily feed intake (ADFI) and feed/gain ratio (F/G) were calculated for each pen. The basal diet was designed to meet feeding standards of pigs (NRC 2012), and the formulation is shown in Table 1. The dietary electrolyte balance (DEB) of the feed was: (1) control group: 96 mmol/kg of the diet for 70–90 kg, 92 mmol/kg of the diet for 90–120 kg; and (2) PMS group: 122 mmol/kg of the diet for 70–90 kg, 118 mmol/kg of the diet for 90–120 kg.

**Figure 1:**
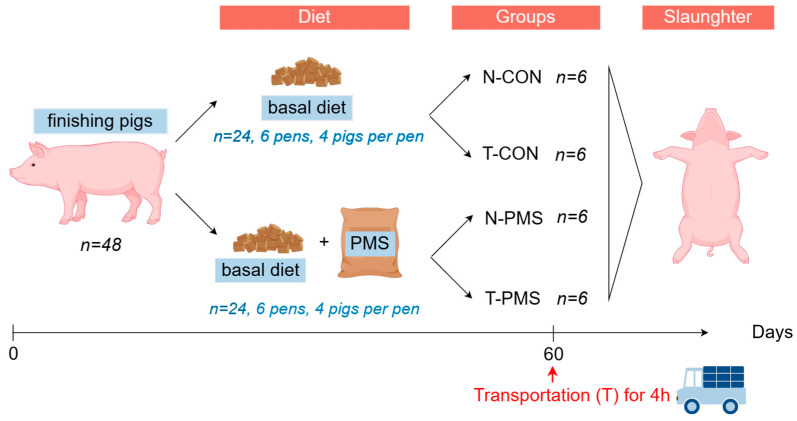
The design of the PMS and transportation treatment of finishing pigs.

The PMS was supplied by Qinghai Lanhu Shancheng Biotechnology Co., Ltd. (Qianghai, China), containing 20% potassium and 6.3% magnesium, and incorporated into a premixed feed. In the second phase, prior to slaughter, two pigs from each pen were randomly selected for slaughter, with one pig undergoing a 4 h transportation stress prior to slaughter. The transportation involved a 300 km journey over 4 h at an average loading density of 200 kg/m^2^, with an ambient temperature of approximately 20 °C. Pigs not subjected to transport were moved from their pens along an aisle and kept in an enclosure for 30 min as the control treatment. Based on diet and transport stress, the slaughtered pigs were categorized into four treatment groups: (1) control group without transport stress, (2) control group with transport stress, (3) PMS-supplemented group without transport stress, and (4) PMS-supplemented group with transport stress. A diagram of the experimental design is shown in Figure 1.

### 2.2. Slaughter and Sampling

The pigs were humanely slaughtered using high voltage electricity (75 V, 1.5 A, 3~4 s). Ten millilitre blood samples were collected from each slaughtered pig, and the serum was isolated via centrifugation at 3000× *g* for 10 min at 4 °C. The serum was subsequently frozen in 1.5 mL centrifuge tubes using liquid nitrogen and stored at −80 °C for later analysis. Following conventional slaughter procedures, the head and hooves were removed, and the liver, heart, kidney, lung, spleen and stomach were excised and weighed. Meat quality traits, including meat colour (measured at 45 min, 24 h, and 48 h post-mortem), drip loss (measured at 24 h and 48 h post-mortem), and shear force were assessed. Jejunal samples were collected from the mid-jejunum, rinsed with ice-cold saline, and stored at −80 °C. Additionally, a 3~5 cm section of the jejunum was preserved in 4% neutral-buffered formalin for intestinal morphology analysis.

### 2.3. Meat Quality Measurement

The longissimus thoracis muscle (LM) from the left side of carcass, devoid of bone and fat, was sampled for meat quality evaluation. The pH of the LM was measured at three different time points using a portable acid meter (testo-205, Testo, Lenzkirch, Germany). Measurements were taken at room temperature for 45 min post-slaughter and after storage at 4 °C for 24 h and 48 h, respectively, by inserting the probe into muscle tissue. The pH value was determined as the average of readings taken at three distinct sites on the muscle. Meat colour parameters, including L* (brightness), a* (redness) and b* (yellowness), were assessed using a Minolta Chroma Meter (CR-410, Minolta, Chiyoda, Japan). Drip loss and shear force were evaluated following the method described previously [18]. Samples were cut into dimensions of 5 cm × 3 cm × 2 cm and then suspended in sealed plastic bags. After storage at 4 °C for 24 and 48 h, the percentage of drip loss was calculated with the samples’ weight before and after the storage.

### 2.4. Serum Parameters Determination

Serum concentrations of epinephrine (EPI), norepinephrine (NE), glucocorticoids (GC), and cortisol (COR) were quantified using ELISA kits, purchased from Shanghai Enzyme-linked Biotechnology Co., Ltd. (Shanghai, China). Antioxidant parameters, including catalase (CAT), total superoxide dismutase (T-SOD), total antioxidant capacity (T-AOC), glutathione peroxidase (GSH-Px), and malondialdehyde (MDA), were assessed using kits from Nanjing Jiancheng Bioengineering Institute (Nanjing, China). Blood biochemical indexes, such as blood glucose (GLU), total protein (TP), albumin (ALB), blood urea nitrogen (BUN), triglyceride (TG), total bilirubin (TBIL), total cholesterol (CHO), high-density lipoprotein cholesterol (HDL-C), low-density lipoprotein cholesterol (LDL-C), alanine aminotransferase (ALT), aspartate aminotransferase (AST), alkaline phosphatase (AKP), and creatinine (CRE) were determined by using kits from BioSino Bio-Technology and Science Inc. with a biochemical analyser (Selectra Pro XL, ELITechGroup, Puteaux, France).

### 2.5. Intestinal Morphology Observation

Intestinal morphology was assessed following the methodology described in a previous study [19]. The jejunum segments were fixed in 10% phosphate-buffered formalin, sectioned to a thickness 5 mm, and stained with hematoxylin and eosin (H&E). Images were captured using a fluorescent proto chromatic difference microscope (Haier, Qingdao, China). For each section, ten regions with optimal visual fields were randomly selected under a light microscope, and measurements of the villus height and crypt depth were performed using Image-Pro Plus software 6.0 (Media Cybernetics, Rockville, MD, USA) [20].

### 2.6. Statistical Analysis

Data are represented as means ± SEM and were analysed using the IBM SPSS Statistics V18.0 software package (IBM Corp., Armonk, NY, USA). For growth performance data, each pen was treated as the experimental unit, and *t*-tests were used for statistical analysis. Figures were drawn by using Figdraw 2.0. For all other datasets, individual pigs were considered as the experimental unit, and statistical analysis was conducted using two-way ANOVA, followed by the least significant difference (LSD) test for post hoc comparisons. Differences were considered significant at a *p*-value of <0.05 and with a significant tendency at 0.05 ≤ *p* < 0.10.

## 3. Results

### 3.1. PMS Supplementation Has No Effect on Growth Performance

As shown in Table 2, PMS supplementation during the fattening stage had no significant effect on growth performance (*p* > 0.05).

### 3.2. PMS Supplementation Mitigates Stress Response Induced by Transport

As indicated in Table 3, dietary supplementation with PMS significantly mitigated the stress response in finishing pigs. Pigs subjected to transportation exhibited higher concentrations of NE (*p* = 0.09) and COR (*p* = 0.04) compared to those in the non-transported group. Notably, PMS supplementation led to a significant reduction in serum COR levels (*p* < 0.05) and showed a trend towards decreasing serum NE levels (*p* = 0.06).

### 3.3. PMS Supplementation Enhances Antioxidant Activity

As illustrated in Table 4, PMS supplementation enhanced antioxidant activity. No significant changes were observed in MDA levels with either PMS supplementation or transportation. However, transportation markedly decreased the serum GSH-Px activity and T-AOC content (*p* < 0.05). Conversely, PMS supplementation significantly increased serum CAT activity (*p* < 0.05) and T-AOC content, and there was a trend towards enhanced GSH-Px activity (0.05 < *p* < 0.1). Additionally, there was a trend suggesting that the interaction between PMS supplementation and transportation elevated serum T-AOC levels (0.05 < *p* < 0.1).

### 3.4. PMS Supplementation Affects Serum Biochemical Profile

As presented in Table 5, transport treatment significantly reduced serum TG levels (*p* < 0.05). Dietary supplementation with 0.5% PMS increased AKP activity (*p* < 0.05), HDL-C levels (*p* < 0.05), and TBIL levels (0.05 < *p* < 0.1), as well as reduced CHO levels (*p* < 0.05) and AST activity (0.05 < *p* < 0.1). Additionally, a potential interaction was observed between PMS supplementation and transportation on serum ALB concentrations (0.05 < *p* < 0.1).

### 3.5. PMS Supplementation Protects Jejunal Morphology

**Figure 2:**
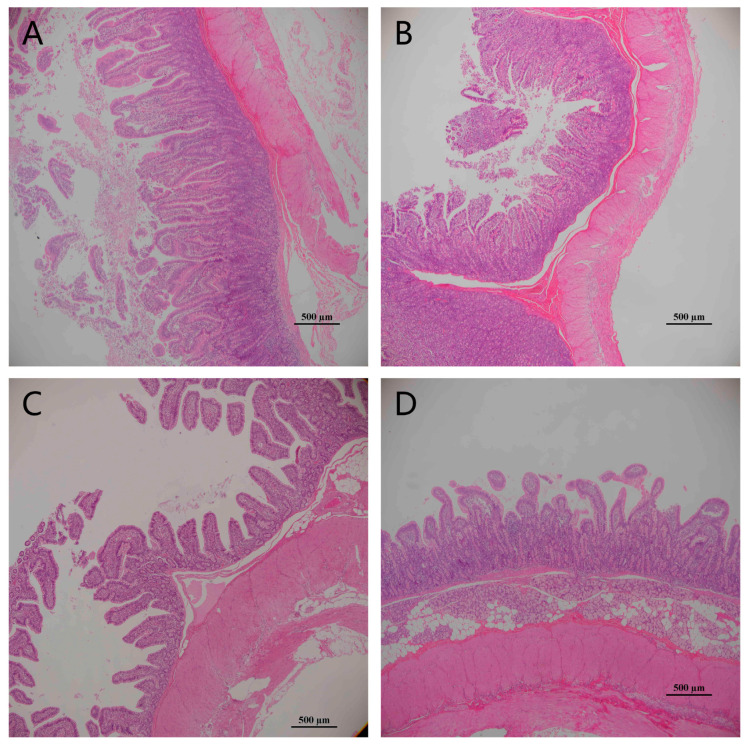
The jejunal morphology of finishing pigs in experimental treatments. (**A**) CON, (**B**) PMS, (**C**) T-CON, (**D**) T-PMS.

Figure 2 and Table 6 indicate that pigs transported for 4 h had a shorter villus height compared to those not transported (0.05 < *p* < 0.1). However, dietary PMS supplementation repaired the intestinal morphological damage caused by transport stress.

### 3.6. PMS Supplementation Improves the Meat Quality

Table 7 presents the effects of PMS and transportation on the meat quality of finishing pigs. The findings reveal that transportation induced a significant decrease in pH_45min_ (*p* < 0.05) and increase in drip loss_24h_ (*p* < 0.05) in the LM. Dietary PMS supplementation showed a trend towards increasing pH_45min_ (*p* = 0.09) and reducing drip loss_24h_ (*p* = 0.09) and drip loss_48h_ (*p* = 0.05). Moreover, PMS supplementation significantly decreased the shear force of the LM (*p* < 0.05). Additionally, PMS supplementation mitigated the increase in 24 h drip loss caused by transport stress.

## 4. Discussion

Finishing pigs are required to be transported by vehicles from the farm to the slaughterhouse. Nevertheless, it has been reported that transport stress imposed on pigs can affect their intestinal tract and meat quality [7,21]. Consequently, it is of great significance to mitigate the harm of transport stress to pigs and preserve the quality of pork. Potassium ions, present at high concentrations (140~160 mmol/L) in all living cells, are crucial for maintaining physiological homeostasis, facilitating transmembrane transport, and supporting nutrient synthesis in animals [22]. Magnesium, as one of the four key elements determining the electrolyte balance in animals, plays a pivotal role in maintaining this balance [23,24]. Potassium and magnesium, as important mineral elements, have been extensively utilized in the production practices of growing and finishing pigs [25,26,27]. In this study, we investigated the potential of dietary PMS supplementation to alleviate the decline in meat quality associated with transport stress in finishing pigs. We conducted laboratory analyses focusing on stress-associated hormones, antioxidant capacity, serum biochemical profile, and meat quality traits. Our results indicated that dietary PMS supplementation exerted beneficial effects in pigs irrespective of transport exposure.

The analysis of serum parameters provides a clear response to disease or physiological changes in animals [2,28,29]. The serum COR concentration, which is a marker for stress development, changes after pigs are subjected to stress [11]. Additionally, serum epinephrine, norepinephrine, and glucocorticoid are critical hormones involved in the stress response in pigs [29]. Our results indicate that transport stress triggers an enhanced stress response, as evidenced by elevated serum norepinephrine and cortisol levels. Supplementation with PMS effectively reduced these elevated levels, suggesting that PMS has a mitigating effect on transport-induced stress.

Malondialdehyde (MDA), a lipid peroxidation product, is a common indicator of oxidative stress, reflecting the extent of lipid peroxidation in cellular membranes [3,30]. MDA can induce cellular damage via peroxidation of polyunsaturated fatty acids or degradation products of lipid peroxides [31]. Antioxidant enzymes such as SOD, CAT, and GSH-Px play vital roles in inhibiting free radicals oxidation and participating in antioxidant defence [6,32,33]. In this study, supplementation with 0.50% potassium magnesium sulphate significantly increased serum CAT activity, with trends towards increased GSH-Px activity and T-AOC. A significant reduction in serum GSH-Px activity was observed following 4 h of transport. Interactions between potassium magnesium sulphate and transport significantly affected T-AOC.

Variations in serum biochemical profiles are closely related to metabolism, nutritional status and disease occurrence. ALB, synthesized by the liver, is associated with the first limiting amino acid, lysine (Lys), in pigs [34]. TG, energy substances with substantial storage and production capacity, are formed by the esterification of three fatty acid molecules with glycerol [35]. Elevated TG levels can independently predict high-density lipoprotein cholesterol (HDL-C), cholesterol transporter activity (CETP), and oxidized low-density lipoprotein (OX-LDL) levels, serving as a decisive factor in atherosclerosis [36]. Serum enzymes such as ALT, AST and AKP are commonly used to reflect liver health and hepatocyte damage [37]. Wang et al. [38] found that the stress of high temperature and humidity may affect serum levels of AKP and TBIL by causing liver damage. In this study, potassium magnesium sulphate supplementation resulted in significant increases in serum AKP activity and HDL-C content, alongside a significant decrease in CHO content. Trends towards reduced AST activity and increased TBIL content were also observed, suggesting potential health benefits of potassium magnesium sulphate supplementation in pigs.

The jejunal villus in the CON were intact, whereas transport-stressed pigs exhibited shorter and more scattered villus, a phenomenon consistent with Zou [2,10]. Other studies have shown that the same situation occurs for the intestinal morphology of fertile pigs after stimulation by high temperature [39]. The present study showed that transport stress adversely affected the intestinal health of finishing pigs, as evidenced by a decrease in intestinal villi height, and this condition could be ameliorated by PMS supplementation.

During the slaughter process, animals experience short-term acute stress, which accelerates muscle metabolism and leads to a rapid decline in muscle pH in the early post-mortem period [40]. This pH reduction occurs because glycogen is converted to lactate and H^+^ via glycolysis [40]. A relatively low muscle glycogen content is believed to limit post-mortem glycolysis and subsequent pH decline [3]. Drip loss, which occurs during the storage period after slaughter, is a crucial indicator of meat quality. The physiological and biochemical reactions in muscles post-slaughter can lead to a decrease in muscle water-holding capacity, increasing drip loss, thereby reducing meat quality and causing significant economic losses. In this study, the addition of potassium magnesium sulphate did not significantly alter muscle pH, but showed a trend towards increased pH_45min_ post-mortem. Transportation markedly decreased pH45_min_ in the LM. Analysis of 48 h drip loss revealed that potassium magnesium sulphate supplementation reduced the water loss, thereby enhancing water retention in pork.

## 5. Conclusions

In conclusion, PMS may be a potential feed additive to improve the pork quality of finishing pigs after stress challenge. More detailed information, such as the mechanism of PMS regulation, need to be further discussed.

## Figures and Tables

**Table 1:** Compositions and nutrient levels of basal diet (%, as dry matter basis).

Ingredients	Body Weight	
	70~90 kg	90~120 kg
Corn	83.08	85.87
Soybean meal	13.00	10.50
NaCl	0.30	0.30
CaHPO_4_	0.95	0.85
Limestone	0.85	0.85
Lysine	0.50	0.40
Methionine	0.09	0.05
Threonine	0.20	0.15
Tryptophan	0.03	0.03
Premix ^a^	1.00	1.00
Total	100.00	100.00
Nutritional levels ^b^		
DE, kcal/kg	3400.00	3400.00
Crude protein, %	13.00	12.00
Lysine, %	0.97	0.83
Methionine + Cysteine, %	0.54	0.50
Threonine, %	0.65	0.56
Tryptophan, %	0.15	0.15
Calcium, %	0.59	0.51
Total phosphorus, %	0.46	0.43
STTD phosphorus, %	0.27	0.25
Potassium, %	0.52	0.48
Magnesium, %	0.13	0.12

^a^ The premix provided the following per kg of diets: 4 500 U vitamin A, 500 U vitamin D, 50 U vitamin E, 1.5 mg vitamin K, 0.16 mg biotin, 0.3 g choline, 1.5 mg folic acid, 5 mg vitamin B_1_, 8 mg vitamin B_2_, 60 mg vitamin B_3_, 24 mg vitamin B_5_, 5 mg vitamin B_6_, 25 mg vitamin B_12_, 150 mg Fe, 10 mg Mn, 16 mg Cu, 0.7 mg I, and 0.3 mg Se. ^b^ Nutrient levels were calculated values.

**Table 2:** Effects of PMS supplementation on growth performance of finishing pigs.

Items	CON	0.50% PMS	*p*-Value
Initial BW, kg	68.30 ± 0.30	68.58 ± 0.09	0.38
Final BW, kg	121.63 ± 1.94	123.85 ± 1.70	0.09
ADG, kg/d	0.89 ± 0.03	0.92 ± 0.03	0.09
ADFI, kg/d	2.91 ± 0.06	2.97 ± 0.06	0.18
F/G	3.29 ± 0.05	3.25 ± 0.05	0.56

Values are presented as mean and pooled SEM, n = 6. Each pen was treated as the experimental unit, and *t*-tests were used for statistical analysis. Differences were considered significantly different at a *p*-value of <0.05 and with a significant tendency at 0.05 ≤ *p* < 0.10. CON, receiving a basal diet; 0.50% PMS, receiving a basal diet with 0.50% PMS supplementation; BW, body weight; ADG, average daily gain; ADFI, average daily feed intake; F/G, feed-to-gain ratio.

**Table 3:** Effects of PMS supplementation and transport on stress-associated hormones of finishing pigs.

Items	N ^a^		T ^a^		SEM	*p*-Value		
	CON	PMS	CON	PMS		PMS	T	PMS × T
EPI (ng/mL)	2.52	1.58	2.80	1.42	0.36	0.13	0.94	0.77
NE (ng/mL)	2.76	1.82	4.29	2.63	0.36	0.06	0.09	0.59
GC (ng/mL)	4.32	3.37	5.56	2.97	0.58	0.13	0.84	0.52
COR (ng/mL)	26.94	24.87	30.01	26.41	0.65	0.02	0.04	0.49

Values are presented as mean and pooled SEM, n = 6. Individual pigs were considered as the experimental unit for the statistical analysis conducted using two-way ANOVA, followed by the least significant difference (LSD) test for post hoc comparisons. ^a^ No transport treatment (N) or transport treatment for 4 h (T). CON, receiving a basal diet; PMS, receiving a basal diet with 0.50% PMS supplementation; EPI, epinephrine; NE, norepinephrine; GC, glucocorticoids; COR, cortisol.

**Table 4:** Effects of PMS supplementation and transport on serum antioxidant activity of finishing pigs.

Items	N ^a^		T ^a^		SEM	*p*-Value		
	CON	PMS	CON	PMS		PMS	T	PMS × T
MDA (nmol/mL)	3.45	3.47	3.59	3.89	0.11	0.47	0.21	0.52
CAT (U/mL)	39.18	86.72	27.10	71.61	8.49	0.01	0.36	0.92
T-SOD (U/mL)	100.47	99.36	92.38	102.14	1.44	0.11	0.31	0.04
GSH-Px (U/mL)	829.31	805.81	704.18	778.80	13.92	0.09	0.02	0.16
T-AOC (mmol/mL)	1.19	1.39	1.11	1.24	0.02	0.00	0.00	0.09

Values are presented as mean and pooled SEM, n = 6. Individual pigs were as considered as the experimental unit in the statistical analysis conducted using two-way ANOVA, followed by the least significant difference (LSD) test for post hoc comparisons. ^a^ No transport treatment (N) or transport treatment for 4 h (T). CON, receiving a basal diet; PMS, receiving a basal diet with 0.50% PMS supplementation; CAT, catalase; T-SOD, total superoxide dismutase; T-AOC, total antioxidant capacity; GSH-Px, glutathione peroxidase; MDA, malondialdehyde.

**Table 5:** Effect of PMS supplementation and transport on serum biochemical profile of finishing pigs.

	N ^a^		T ^a^		SEM	*p*-Value		
	CON	PMS	CON	PMS		PMS	T	PMS × T
TP (g/L)	65.87	66.16	65.12	69.11	0.96	0.28	0.58	0.35
ALB (g/L)	38.87	32.31	30.17	34.63	1.42	0.70	0.25	0.06
BUN (mmol/L)	1.84	1.99	1.86	2.62	0.15	0.14	0.28	0.31
AST (U/L)	61.26	35.95	57.43	51.05	4.06	0.05	0.47	0.23
ALT(U/L)	51.18	42.89	46.04	52.51	2.55	0.86	0.67	0.18
TBIL (μmol/L)	4.46	5.46	4.00	4.73	0.23	0.07	0.20	0.77
CRE (μmol/L)	152.73	149.16	147.31	144.25	4.29	0.72	0.58	0.98
AKP (U/L)	90.42	120.21	108.53	125.93	5.53	0.03	0.26	0.55
GLU (mmol/L)	6.35	5.67	6.74	6.59	0.26	0.45	0.24	0.63
TG (mmol/L)	0.48	0.84	0.32	0.39	0.72	0.11	0.03	0.27
CHO (mmol/L)	2.44	2.07	2.20	2.02	0.67	0.04	0.27	0.46
HDL-C (mmol/L)	0.79	0.98	0.88	0.99	0.10	0.01	0.44	0.45
LDL-C (mmol/L)	1.00	0.91	0.83	1.05	0.06	0.59	0.90	0.19

Values are presented as mean and pooled SEM, n = 6. Individual pigs were considered as the experimental unit for the statistical analysis conducted using two-way ANOVA, followed by the least significant difference (LSD) test for post hoc comparisons. ^a^ No transport treatment (N) or transport treatment for 4 h (T). CON, receiving a basal diet; PMS, receiving a basal diet with 0.50% PMS supplementation; TP, total protein; GLU, blood glucose; ALB, albumin; BUN, blood urea nitrogen; TG, triglyceride; TBIL, total bilirubin; CHO, total cholesterol; HDL-C, high-density lipoprotein cholesterol; LDL-C, low-density lipoprotein cholesterol; ALT, alanine aminotransferase; AST, aspartate aminotransferase; AKP, alkaline phosphatase; CRE, creatinine.

**Table 6:** Effect of PMS supplementation and transport on the jejunal morphology of finishing pigs.

Items	N ^a^		T ^a^		SEM	*p*-Value		
	CON	PMS	CON	PMS		PMS	T	PMS × T
Villus height (μm)	515.53	518.05	404.10	508.01	19.61	0.12	0.08	0.14
Crypt depth (μm)	281.89	303.58	287.08	299.16	13.70	0.42	0.82	0.96

Values are presented as mean and pooled SEM, n = 6. Individual pigs are considered as the experimental unit for the statistical analysis conducted using two-way ANOVA, followed by the least significant difference (LSD) test for post hoc comparisons. ^a^ No transport treatment (N) or transport treatment for 4 h (T). CON, receiving a basal diet; PMS, receiving a basal diet with 0.50% PMS supplementation.

**Table 7:** Effects of PMS supplementation and transport on the meat quality traits of finishing pigs.

Items	N ^a^		T ^a^		SEM	*p*-Value		
	CON	PMS	CON	PMS		PMS	T	PMS × T
L*_45 min_	51.29	52.09	51.41	51.79	0.39	0.49	0.92	0.81
a*_45 min_	17.64	16.59	17.34	17.04	0.18	0.06	0.84	0.28
b*_45 min_	7.85	7.84	7.86	7.84	0.46	0.43	0.43	0.43
L*_24 h_	58.58	60.99	59.38	59.20	0.55	0.34	0.67	0.27
a*_24 h_	19.04	19.18	17.99	18.11	0.44	0.89	0.27	0.99
b*_24 h_	11.96	12.88	11.60	11.72	0.57	0.67	0.54	0.74
L*_48 h_	59.10	60.92	59.72	60.28	0.51	0.28	0.99	0.56
a*_48 h_	17.48	16.91	17.25	17.40	0.23	0.68	0.79	0.48
b*_48 h_	9.02	9.26	9.12	9.06	0.18	0.81	0.89	0.70
pH_45 min_	5.99	6.06	5.69	5.95	0.05	0.09	0.04	0.34
pH_24 h_	5.56	5.53	5.52	5.49	0.02	0.34	0.30	0.86
pH_48 h_	5.26	5.19	5.27	5.24	0.02	0.16	0.32	0.49
Drip loss_24 h_ (%)	3.26	2.39	3.67	3.39	0.18	0.09	0.04	0.38
Drip loss _48 h_ (%)	4.48	3.52	3.94	3.69	0.15	0.05	0.54	0.24
Shear force (N)	71.31	52.64	64.81	59.84	2.71	0.03	0.94	0.19

Values are presented as mean and pooled SEM, n = 6. Individual pigs are considered as the experimental unit for the statistical analysis conducted using two-way ANOVA, followed by the least significant difference (LSD) test for post hoc comparisons. ^a^ No transport treatment (N)or transport treatment for 4 h (T). CON, receiving a basal diet; PMS, receiving a basal diet with 0.50% PMS supplementation.

## Data Availability

The original contributions presented in this study are included in the article. Further inquiries can be directed to the corresponding authors.
